# Telehealth sounds a bit challenging, but it has potential: participant and physiotherapist experiences of gym-based exercise intervention for Achilles tendinopathy monitored via telehealth

**DOI:** 10.1186/s12891-020-03907-w

**Published:** 2021-02-04

**Authors:** F. Hasani, P. Malliaras, T. Haines, S. E. Munteanu, J. White, J. Ridgway, P. Nicklen, A. Moran, P. Jansons

**Affiliations:** 1 Physiotherapy Department, School of Primary and Allied Health Care, Monash University, Frankston, Victoria 3199 Australia; 2 Physiotherapy Department, Security Forces Hospital, Riyadh, 11481 Kingdom of Saudi Arabia; 3 School of Primary and Allied Health Care, Faculty of Medicine, Nursing, and Health Sciences, Monash University, Frankston, Victoria 3199 Australia; 4 Discipline of Podiatry, School of Allied Health, Human Services and Sport, College of Science, Health and Engineering, La Trobe University, Melbourne, Victoria 3086 Australia; 5 La Trobe Sport and Exercise Medicine Research Centre, School of Allied Health, Human Services and Sport, College of Science, Health and Engineering, La Trobe University, Melbourne, Victoria 3086 Australia; 6 Research Centre for Generational Health and Ageing, School of Medicine and Public Health, University of Newcastle, Callaghan, NSW 2308 Australia; 7 Physiotherapy Department, Peninsula Health, Frankston, Victoria 3199 Australia; 8 Back in Motion Physical Therapy, Melbourne, Victoria 3195 Australia; 9 Department of Medicine, School of Clinical Sciences at Monash Health, Monash University, Clayton, Victoria Australia; 10 Institute for Physical Activity and Nutrition (IPAN), School of Exercise and Nutrition Sciences, Deakin University, Geelong, Australia

**Keywords:** Achilles tendinopathy, Exercise, Telehealth, Barriers, Enablers, Satisfaction, Qualitative

## Abstract

**Background:**

Although telehealth is becoming more popular for delivery of care for individuals with musculoskeletal pain, to our knowledge telehealth has not been used to manage Achilles tendinopathy. This research aimed to explore the experience of participants and physiotherapists with gym-based exercise interventions for Achilles tendinopathy monitored via videoconference.

**Methods:**

A qualitative, interpretive description design was performed using semi-structured interviews (8 participants) and a focus group (7 physiotherapists). Participants and physiotherapists were interviewed about their experiences of the use of telehealth during a gym-based exercise intervention incorporating different calf load parameters for Achilles tendinopathy. We employed an inductive thematic analysis approach to analyse the data.

**Results:**

Three themes identified from both participants and physiotherapists included i) acceptability of telehealth; ii) enablers to adherence with telehealth; and iii) barriers to adherence with telehealth. Two extra themes arose from participants regarding adherence with gym-based exercise, including enablers to adherence with the exercise intervention, and barriers to adherence with the exercise intervention. Both participants and physiotherapists expressed overall satisfaction and acceptability of telehealth monitoring of gym-based exercise.

**Conclusion:**

Gym-based exercise intervention for Achilles tendinopathy involving weekly telehealth monitoring was acceptable to both participants and physiotherapists. Potential enablers and barriers were identified that may improve adherence to this type of intervention.

**Supplementary Information:**

The online version contains supplementary material available at 10.1186/s12891-020-03907-w.

## Background

Mid-portion Achilles tendinopathy is a highly prevalent musculoskeletal condition, affecting both athletes and the general population [1, 2]. The condition is characterised by persistent, activity-related pain and impaired function such as walking and running [3]. Exercise-based interventions that aim to restore tendon loading capacity using progressive loading are recommended as a first line treatment in clinical practice guidelines for the management of Achilles tendinopathy [4].

Although exercise is promoted as an evidence-based treatment, there is no clear research guidance about which exercise approach is optimal [5, 6], and there is limited understanding of the exercise parameters that may confer the greatest benefit [6, 7]. Recent systematic reviews reported insufficient information to replicate the exercise intervention or determine the prescribed dose [6, 7]. For instance, only one of the 24 exercise trials (4%) specified the magnitude of load used during calf muscle exercise. In addition, only 58% of the trials measured adherence, and it was assessed via self-report which may be prone to recall bias if participants need to recall over longer periods [6]. In response, we undertook a feasibility trial to compare the efficacy of different loading parameters (load-intensity or time-under-tension on clinical outcomes among individuals with Achilles tendinopathy [8]. The exercise intervention was monitored using videoconferencing and was undertaken by participants in their local gym environment. We hypothesised that exercise adherence and fidelity would be optimised using a weekly telehealth session with a physiotherapist. Although telehealth is becoming more popular for delivery of care for people with musculoskeletal pain [9, 10], to our knowledge telehealth has not been used to manage Achilles tendinopathy.

Proposed advantages of telehealth include convenience for both participants and physiotherapists and improved equity of access to healthcare [11]. However, there are potential concerns about whether telehealth is acceptable to participants and physiotherapists, for example, if they perceive it can be an appropriate replacement for face-to-face care [12, 13]. A lack of knowledge and experience using telehealth in clinical practice by physiotherapists has been identified as a potential challenge with successful implementation [9].

Qualitative techniques are used to explore research questions inductively in natural contexts. Qualitative research enables participants to provide a detailed account of their experiences and present their own perspectives and interpretation of these experiences [14], which cannot always be captured using quantitative methods [15]. This qualitative study was nested within a pilot randomised trial [8] and aimed to explore the experience of participants and physiotherapists with gym-based exercise interventions for Achilles tendinopathy with weekly telehealth monitoring (via videoconference). Understanding stakeholder views may help to develop acceptable and effective telehealth interventions for Achilles tendinopathy.

## Methods

### Study design

This qualitative study was nested within a four-arm, factorial randomised pilot study (Monash University ethics approval: 2018–1366-20,711) investigating the efficacy of high or low-intensity exercise, performed with either high or low time-under-tension, for the management of Achilles tendinopathy. The protocol was registered at the Australian New Zealand Clinical Trials Registry (06/08/2018, ACTRN 12618001315202). There were 12 participants per factorial arm. The trial protocol has previously been described [8]. Recruitment occurred between April and July 2019 in an Australian setting. All participants provided consent to participate in a qualitative interview. The study was reported in accordance with the consolidated criteria for reporting qualitative research [16].

### Participants

#### Participants with Achilles tendinopathy

Potential participants were purposively sampled from the broader study [8]. In order to gain a wide range of perspectives, we invited participants from each of the four factorial groups who exhibited a range of self-reported primary outcome scores at final follow-up (12 weeks), and demonstrated acceptable and unacceptable adherence with the exercise intervention (acceptable adherence defined as achieving 66% or more of the number of sessions prescribed) [8]. To reduce recall bias, participants meeting inclusion criteria, were contacted within 3 weeks of completing the trial to make an interview time [8].

#### Physiotherapists

Qualified and practicing physiotherapists were recruited (via social media) to deliver the trial interventions (*n* = 8). Physiotherapists who monitored the trial interventions were invited to participate in a focus group, forming a convenience sample (*n* = 7).

### Trial interventions

Prior to the commencement of the trial, each physiotherapist participated in three 1.5 h face-to-face or telehealth training sessions and was provided with multimedia materials (print and video) about the intervention. Physiotherapists were also trained in addressing potential barriers to exercise adherence such as fear-avoidance and pain catastrophising) using a motivational interviewing approach (i.e. understanding the participants perspective, provide appropriate education and advice to empower the participant).

The intervention was a gym-based exercise program where the participants performed 4 sets of unilateral isotonic standing and seated calf raise exercise in a Smith machine (both sides, one leg at a time) three times per week, over 12 weeks. The exercises incorporated different calf load parameters, based on group allocation, including high and low intensity with high and low time-under-tension (i.e. 6 and 18 repetitions per set prior to failure with 2 or 6 s repetitions, respectively). To facilitate adherence, physiotherapists supervised one session per week via videoconference software (Zoom®) that was downloaded to the participants’ smartphone. One physiotherapist was allocated to provide care for each participant. Two physiotherapists were available as a substitute care provider when needed if a physiotherapist was unavailable. Each telehealth session was 30 to 50 min in length, and during the sessions the physiotherapist provided feedback related to exercises technique and progressed training load. At the face-to-face baseline assessment, participants were provided with an inexpensive tripod and trained in how to set-up the telehealth environment, including video settings and camera angle, which was extensively piloted by the research team prior to commencing the trial. Assessing calf raises exercise fidelity has been demonstrated to be reproducible (Hasani et al. *in press).*

### Data collection

#### Qualitative interviews with participants

Participant interviews were conducted by telephone by one physiotherapist researcher who was unknown to the participants. The interviewer undertook training (by PM) in conducting interviews prior to data collection and carried out practice interviews. A semi-structured topic guide (Additional file 1) was used to guide interviews. The interview explored participants experiences with the intervention and telehealth (e.g. what they felt was good and bad) and barriers and enablers to gym-based exercise adherence and adherence with telehealth monitoring. The semi-structured nature permitted flexibility for participants to explore topics that would not have otherwise surfaced [17]. We also collected demographic data (age), history of telehealth use, adherence with the prescribed exercise intervention, disease-status of the participants (self-reported pain and function at baseline using the Victorian Institute of Sport Assessment – Achilles questionnaire (VISA-A) [18] and duration of symptoms), as well as the place of residence (postcode).

#### Focus group among physiotherapists

A moderator and experienced qualitative researcher (JW) conducted the focus group, using telehealth software, and one facilitator (FH) took detailed notes to supplement the recording and support data analysis. A semi-structured schedule of questions guided the focus groups (Additional file 2). The moderator was not involved with the associated implementation of the study; this assisted to reduce bias and facilitated open discussion that allowed physiotherapists to express their opinions.

We collected history of telehealth use of physiotherapists, city of residence, and work-related data for the physiotherapists (work setting, type of employment, number of ankle participants per month).

### Data analysis

Interviews and focus groups were audio-recorded and transcribed verbatim. The transcripts were checked for accuracy against the original recording. Data were analysed using an inductive thematic analysis approach [19]. This involved (i) familiarisation and identifying codes of meaning by reading the transcripts line-by-line, (ii) grouping codes into categories to assist with data retrieval using Microsoft Excel spreadsheets, and (iii) examining relationships between codes based on connections and similarities to form themes. Data were analysed within and between the intervention groups by two independent researchers (FH and PJ). Any differences in researcher-perspective were resolved by negotiation in multiple meetings. The research team (FH, PJ, PM, JW) discussed and refined the final themes in the context of the research question. To increase the credibility of our data, strategies such as peer debriefing and reflexive analysis were used [20]. The number of participants interviewed was based on data sufficiency (judged by reviewing transcripts after each interview and discussion between the researchers). The participants did not have an opportunity to provide feedback on the findings.

Although our objective was not to quantify participants responses, we have used the terms “all” (8 participants); “nearly all” (7 participants); “several” (6–3 participants); and “a few” (≤ 2 participants) to provide the readers with an indication of the frequency of agreement of each theme and sub-themes. Divergent views were reported within the themes. The nature of the focus group did not allow us to quantify the frequency of themes expressed among the physiotherapists.

## Results

Data from eight participants (two from each factorial arm) and seven of the eight physiotherapists who monitored interventions were analysed. One physiotherapist (based on Spain) was unable to participate in the focus groups due to schedule conflict. Participants with Achilles tendinopathy were aged between 38 to 54 years and baseline VISA-A scores ranged from 17 to 74 (out of 100, with higher scores indicating more severe pain and impaired function). The interviews ranged from 20 to 30 min in length. Participants characteristics are presented in Table 1. Six physiotherapists were based in Australia and one in South Africa. Physiotherapists’ work experience ranged from 3 to 22 years (Table 2). The physiotherapist focus group meeting lasted for 60 min.

**Table 1:** Characteristics of participants with Achilles tendinopathy

ID	Group	Age (years)	Duration of symptoms (months)	BMI	Employment	Hold gym membership	Previous Zoom use	Tendon pain before intervention ^**a**^	Tendon pain after intervention ^**a**^	Zoom sessions completed (12)	Home sessions completed (36)	Adherence %
1	HL,HT	54	48	27	Full-time	No	Yes	17	41	12	30	83
2	HL,HT	41	7	28	Full-time	No	No	40	92	10	23	64
3	HL,LT	53	240	41	Full-time	No	Yes	23	85	9	16	44
4	HL,LT	38	36	30	Part-time	No	No	74	97	9	21	58
5	LL, HT	51	12	33	Part-time	Yes	No	70	100	10	26	72
6	LL, HT	44	24	25	Full-time	No	No	63	63	11	30	83
7	LL,LT	41	12	22	Full-time	No	No	58	90	12	34	94
8	LL,LT	47	3	20	Full-time	Yes	Yes	53	95	11	21	58

*Abbreviation*: *HL,HT* High intensity with high time-under-tension, *HL,LT* High intensity with low time-under-tension, *LL,HT* Low intensity with high time-under-tension, *LL,LT* Low intensity with low time-under-tension; ^a^Measured by the Victorian Institute of Sports Assessment – Achilles questionnaire (VISA-A) with 100 considered full Achilles tendon health

**Table 2:** Characteristics of physiotherapists

ID	Gender	Age	Clinical experience (years)	Work setting (Private/Public)	Employment	Consults of participants with ankle complaints (number per month)	Previous Zoom use	Residence
1	Female	39	18	Public	Full-time	30	No	Australia
2	Female	36	13	Private	Full-time	10	No	Overseas
3	Male	29	7	Private	Part-time	15	Yes	Australia
4	Male	44	22	Private	Part-time	20	Yes	Australia
5	Male	36	13	Private	Full-time	10	No	Australia
6	Male	30	8	Private	Part-time	30	No	Australia
7	Male	25	3	Private	Part-time	8	Yes	Australia

### Participants findings

**Fig. 1:**
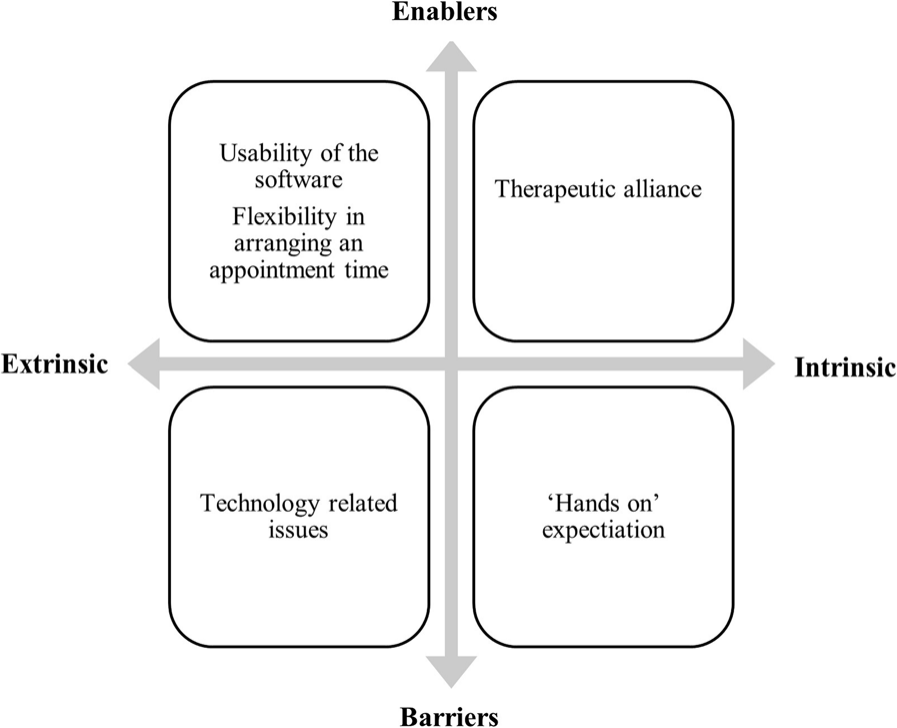
Enablers and barriers to participants’ adherence with telehealth monitoring

**Fig. 2:**
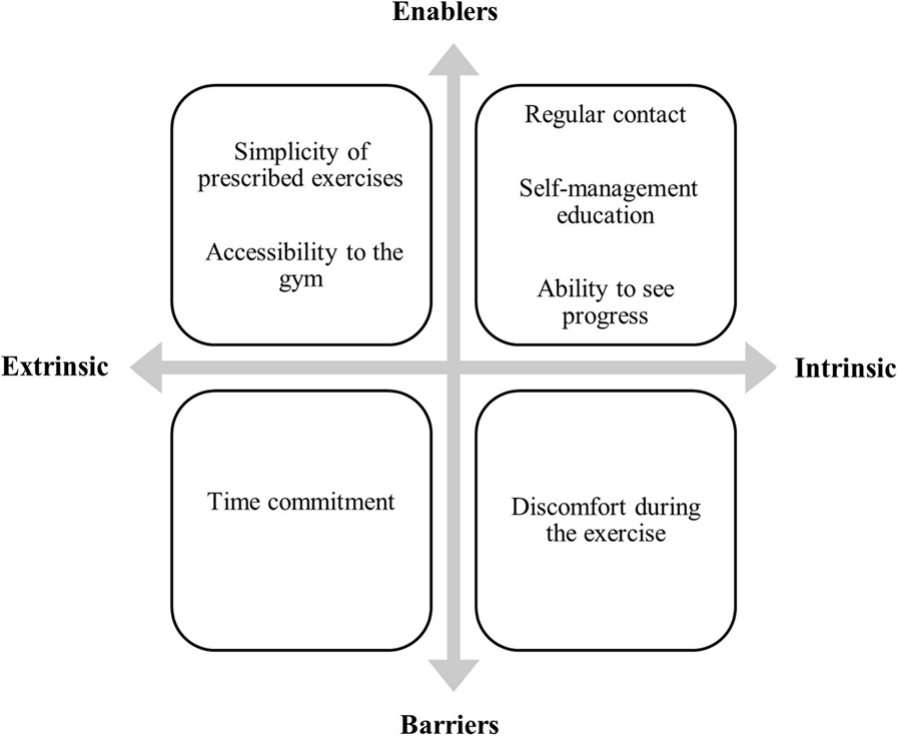
Enablers and barriers to participant adherence with the exercise interventions

Five key themes identified from the analysis of the semi-structured interviews. The themes inform understanding the experience of participants in performing gym-based exercise intervention involving a weekly telehealth monitoring session (Figs. 1 and 2).

#### Theme 1: acceptability of the telehealth sessions and gym-based exercise intervention

All participants [8/8] expressed satisfaction with the quality of service they received via telehealth mainly because it was perceived to be efficient. Participants appreciated the convenience of not having to travel to a clinic and wait for long periods to be seen.

*“I did not have to go and wait in an office or whatever. I could just go to the gym and just get started and if he was not ready or whatever then I could just get into my workout and he [physiotherapist] would join me halfway through sometimes. There was no waiting around.”* (Participant [P] 4).

A few participants [2/8] felt that telehealth suited conditions where ‘hands-on’ assessment was not essential such as after a diagnosis had been determined or when ‘hands on’ therapy such as massage was not required.

*“I guess, there is a certain amount of hands-on with the physio, but depending on the type of issues that someone has, there is certainly scope for being able to treat them from afar, once you have got an accurate idea of what you want to do with them.” (*P 8)

*“If you do not need actual manipulation, like a massage or something like that, or physical touching of the area, then that would be fine.” (*P 7)

All participants [8/8] found progressive loading exercises to be an effective form of treatment compared to their experience of previous treatment approaches.

*“The idea that it is more about of putting it [Achilles tendon] under load for longer has been a lot more effective than all the stretching, warming and icing and everything else that I have tried before.” (*P 8)

#### Theme 2: enablers of adherence with telehealth monitoring

Participants reported common enablers to the telehealth sessions which were grouped into subthemes of usability of the software, flexibility in arranging an appointment time, and therapeutic alliance.

##### Usability of the software

Nearly all participants [7/8] reported that the telehealth software was easy to use. They were positive about how easy it was to install and use the application.

*“The physio would send me a link; I would click on the link on my phone and away [we]-go. It was pretty effortless.”* (P 5)

##### Flexibility in arranging an appointment time

The flexibility and convenience of telehealth allowed participants to schedule treatment/exercise within convenient times that avoided peak gym-use times.

*“Because I am a shift worker and I had really odd hours, it made access to the gym at times pretty good because there was nobody else there.”* (P 1)

##### Therapeutic alliance

All participants [8/8] acknowledged that despite not being face-to-face with their physiotherapist, they still felt reassured by the telehealth contact with their physiotherapist, especially in the early stages when they were gaining familiarity and confidence with the treatment process.

*“She [physiotherapist] was patient and did not treat me like an idiot when I did not know what I was doing in the beginning and things like that … She talked me through it [exercise set up] well and all that, made it easy to understand. She let me know what I was doing at every stage.”* (P 2)

*“They [physiotherapists] were there to say, ‘See what you can do and push yourself as much as you can’, which is good.”* (P 6)

All participants [8/8] reported that telehealth was motivating and promoted commitment to their exercise program.

*“The audio feedback and them [physiotherapists] telling you to go longer, faster, shorter, harder, whatever their instruction happens to be, that is sort of invaluable.”* (P 3)

#### Theme 3: barriers to adherence with telehealth monitoring

Although the technology was reported as having advantages, some barriers were noted that impacted the sessions. Several participants [4/8] complained about poor or no Wi-Fi at their gym. There were reservations about having to use their personal phone internet data for the videoconference call. On several occasions, participants reported they were running out of phone battery which impacted the length of the session.

*“It was the longer regimen that I was on [low-intensity group]. A couple of times my battery went out and I was using up all my data because it was an hour and a half session.”* (P 8)

For a few participants [2/8], assistance was needed to set up the correct camera angle of their phone while they were training on the Smith machine. Guidance from their physiotherapist assisted to overcome this issue.

*“It was more the Smiths machine’s limitations in regard to getting the right height for the phone.”* (P 7)

#### Theme 4: enablers to adherence with the exercise interventions

Participants reported potential enablers to adherence with exercise and these were grouped into subthemes including regular contact, the nature of the exercise, *accessibility to the gym*, *self-management* education, and ability to see progress.

##### Regular contact

Participants appreciated the ability to have regular contact with a physiotherapist for support and obtain immediate feedback to allow them to progress their exercises.

*“I think if you did not have to talk to anybody, you might get lazy some days and not bother. Whereas that sort of kept you committed and making sure you will not forget your exercises.”* (P 6)

##### The nature of the exercise

Several participants [5/8] appreciated the simplicity (i.e. two calf raises exercises) and how clearly they were explained as part of the trial intervention.

*“It was simple and easy to do and straight forward.”* (P 4)

##### Accessibility to the gym

Several participants [5/8] accessed a 24-h gym in a convenient location which reportedly assisted with adherence and motivation.

*“ Access to the 24-hour gym was handy for me, because I work in different hours and have got family and young kids, so it’s quite good that I could go at any time I wanted … and having the various gyms and having access to those around the place definitely helped me, because like I said, I travel a fair bit for work, so that was very handy.”* (P 7)

##### Self-management education

All participants [8/8] noted that the one-to-one self-management education they received at the start of the rehabilitation program was helpful and changed the way they perceive their Achilles symptoms. Specifically, the education improved their self-efficacy and ability to understand acceptable pain limits during activity.

*“I am not worried that I am going to hurt it if I run or anything like that. I know that if I give it a bit of pain it may help it, whereas before I was picturing it sort of snapping and I was going to be in all sorts of trouble and roll it up in my leg or something. Whereas now I do not have that fear.”* (P 2)

*“Having a little bit of pain does not mean to say that you should not be doing the exercises. So as long as it is not getting really bad, that guidance on how to manage the pain aspect of it now that I am thinking about it, was very handy because it gives you framework to decide well is this normal aches and pains or is it something a bit more serious.”* (P 5)

Several participants [6/8] also stated that the education material (booklet) was beneficial when they were exercising on their own. The booklet helped them to self-progress their exercises and review the correct technique.

*“The booklet outlined with photos, the correct and not correct techniques, and what you should and shouldn’t be doing … the actual manual showing me the techniques and stuff was a good thing.”* (P 7)

##### Ability to see progress

All participants [8/8] described that the care provided was effective in terms of improvement in symptoms and function. Being able to observe progress and experience less pain was reportedly a key motivator.

*“I do not have the pain that I used to. I was getting to the stage at times I was quite immobile at work. But I have not had that ever since the program finished … which is great.”* (P 1)

#### Theme 5: barriers to adherence with the exercise interventions

Potential barriers to exercise interventions reported by participants were related to the time and the nature of exercise.

##### Time

Making time to commit to regular exercise was reported as a barrier by several participants [6/8]. This was commonly because of competing commitments e.g. employment or caregiving.

*“Literally just time allocation in what I do. Because I do not work for somebody, so I do not have a nine to five job. I can work twenty-four hours a day. In fact, many days I do. So being able to commit to time in advance is very, very difficult for me.”* (P 3)

Participants [3/8] who performed the low-intensity exercise (18 repetition maximum, which involved 18 rather than 6 repetitions per set) expressed greater concerns about the time commitment required.

*“It was so time-consuming … I did not have much time to do anything else in the gym.”* (P 5)

##### Exercise

Several participants [5/8] expressed a feeling of leg discomfort from the pressure of the Smith bar on their thigh during their seated calf raises.

*“It was suggested that I use like a foam mat or something, but I was up to about 100 odd kilos, it was quite painful to have the bar on.”* (P 5)

### Physiotherapist findings

**Fig. 3:**
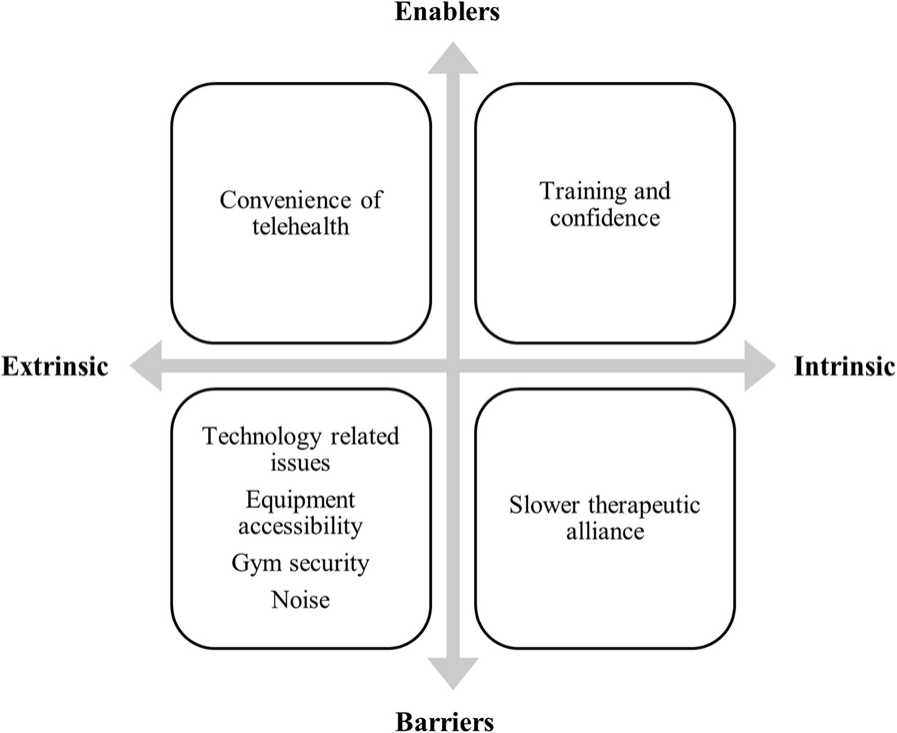
Enablers and barriers to physiotherapists’ adherence with telehealth monitoring

Overall, there were 3 key themes identified to inform the understanding of physiotherapists experience with providing the telehealth sessions as part of exercise-based intervention (Fig. 3).

#### Theme 1: acceptability of the telehealth

All physiotherapists reported satisfaction with the quality of telehealth service they were able to provide. They felt empowered by the role they played in the study and this promoted feelings of confidence in telehealth.

*“I have used the research concepts to improve the telerehab that I do in the clinic … it was much more vigorous and a bit more standardised [than] what we did so I found it very satisfying and I think I have got more confidence.”* (physiotherapist [PT] 4)

#### Theme 2: enablers to telehealth monitoring

Physiotherapists reflected on the accessibility and convenience of telehealth. Further, physiotherapists felt telehealth enabled more regular contact with participants. This was reported to be a source of therapeutic satisfaction as they were able to provide regular reassurance to assist participants’ progress.

*“I had one [participant] who was fly out working in WA and it was great that we Zoomed.”* (PT 6)

*“I found that close contact especially with quite anxious type [participants ], just being able to reassure them that where their early stages were absolutely fine and appropriate and to be expected, I think that helped them stick to the course.”* (PT 4)

Consistent with this, physiotherapists proposed that more encouragement and motivation was needed for participants requiring longer time to complete their sessions (i.e. participants in low-intensity groups who completed 18 repetitions per set rather than six). One of proposed strategy was a follow up email post session.

*“I think other people who got irritated around time at the gym they definitely did longer session … I liked the follow up emails after the session, sort of template kind of idea of what you could send them [to participants ] at some point positive things from their session encouraging them for the next week.”* (PT 3)

Physiotherapists also reported that having substitute care provider, who was available to cover their sessions when it was needed, reduced the stress associated with solving booking issues.

*“Working in a private practice can be difficult to take time off randomly and I think (the substitute physiotherapist) has been fantastic at that often supports and stepped in.”* (PT 5)

Physiotherapists reported that training prior to commencement of the trial was valuable and clarified questions about their role in the research. The training improved their confidence in being able to deliver the intervention via telehealth.

*“I think we [as physiotherapists] got a lot of information prior the trial so for me all the documents that we received actually allowed the process to be very routine and very kind of straight forward and I think obviously once you have done one or two sessions it really starts to become just quite mechanically because you know what you are doing and you know what your expectations are.”* (PT 2)

#### Theme 3: barriers to telehealth monitoring

Key barriers reported by physiotherapists related to technology, the therapeutic *alliance* and the gym-based exercise.

##### Technology-related

Physiotherapists noted multiple technology-related issues during the telehealth sessions including poor videoconference quality or loss of videoconference connection, insufficient participant phone battery life and inability to operate the videoconference video function. Some sessions had to be discontinued due to technology-related issues.

*“Sometimes technical issues and connectivity sort of problems … a couple of times participants have forgot to recharge their phone properly and phone might have sort of drained out.”* (PT 5)

A few physiotherapists discussed the limitations of only having one camera angle to view participants and assist with providing feedback related to calf exercise fidelity. In some cases, physiotherapists experimented with camera angles to improve visualisation.

*“I am not entirely sure of how easy that is [telehealth] to correct technique rather than being there in person to observe that in more detail we see from different angles and adjust things as you are going so.”* (PT 1)

##### Therapeutic alliance

Although physiotherapists reported that the use of telehealth was an effective method for therapeutic interactions, they also reported some barriers to developing a therapeutic alliance.

*“I was probably a little slower to develop the relationship, so I found a couple of sessions to sort of warm up and get a bit more hold over messages.”* (PT 1)

##### Gym-based exercise

Physiotherapists reported some challenges accessing the Smith machine for some of their participants especially when the local gym had limited equipment (e.g. only one Smith machine). In some cases sessions had to be rescheduled due to long waiting times.

*“ I think most people have that gym etiquette that someone is using [the machine] then leave them alone and then just keep the workout or do something else, but couple of times I have had to cancel a session because patients could not access the Smith machine for like half an hour or 40 minute.”* (PT 5)

Physiotherapists reported that they needed to justify the telehealth monitoring (i.e. that they were part of a study) to gym staff. Some of the prescribed gym exercises were not conventional (e.g. performing seated calf raise exercise on a Smith machine without shoes on) and at times this required negotiation with gym staff (e.g. whether it was possible to exercise with shows off).

*“I thought that they might feel a bit self-conscious being at the gym and chatting away, but most of them wholeheartedly just come and had no issue whatsoever with doing it, so that was good … but I had a couple of the gyms that did not enjoy the patients having their shoes off during the sessions so we had a number of those where either negotiate with the gym or they [patients ]had to wear different shoes.”* (PT 2)

Even though participants wore Bluetooth headsets during telehealth sessions, noise from background music or other gym users was reported by physiotherapists to have been a distraction although it was manageable.

*“There were a few minor issues like the music in background but nothing that actually stop us from doing any sessions.”* (PT 4)

## Discussion

This study explored the experience of participants and physiotherapists with gym-based exercise interventions for Achilles tendinopathy that was monitored weekly via telehealth. Our findings provide valuable anthropological information related to acceptability, barriers and enablers related to both the gym-based exercise intervention and telehealth monitoring that may inform future research and clinical practice. Participants reported usability of the telehealth software and flexibility in scheduling appointment times to be extrinsic enablers and therapeutic alliance with physiotherapists as an intrinsic enabler. Technical issues associated with technology such as internet connectivity and phone battery were the key extrinsic barriers to telehealth monitoring. Whereas physiotherapists reported challenges associated to technology (e.g. poor internet quality, insufficient participant phone battery life and inability to operate the videoconference video function) as well as to the gym environment (e.g. accessing the Smith machine, noise and restrictive gym rules) as barriers. Being able to access and therefore influence participants more regular was a key intrinsic enabler related to telehealth monitoring for the physiotherapists. Extrinsic enablers to the exercise interventions reported by participants included the simplicity of prescribed exercises and the accessibility of the gym. Intrinsic enablers included regular contact, self-management education, and the ability to see progress. The time commitment was reported to be a key extrinsic barrier that may have diminished the participations’ adherence. These themes will be discussed here in the context of existing literature and suggestions for future studies will be provided.

### Telehealth was acceptable

An important perspective apparent in this research was the overall satisfaction and acceptability of telehealth intervention to manage the pain and dysfunction associated with Achilles tendinopathy. This acceptance among participants and physiotherapists reinforces the hypothesis that the delivery of rehabilitative services at a distance is feasible [21]. Based on our findings, the drivers underlying this acceptance appear to be the time efficiency and flexibility of telehealth. To the best of our knowledge, there are no previous trials using a similar delivery mode as an intervention for individuals with Achilles tendinopathy to directly compare our findings. However, consistent with our findings, sedentary middle-aged adults with knee osteoarthritis have expressed acceptance of telehealth delivered, physiotherapist-prescribed home exercise or education. Specifically, participants involved in the telehealth arm of this trial reported that the intervention was flexible, time efficient and improved access [22].

### Telehealth barriers and enablers

Adherence to the weekly telehealth monitoring sessions was high, ranging from 71 to 92%. This may be partly explained by some of the enablers reported by participants. Participants felt Zoom was a user-friendly videoconference software. Using a freely available and simple videoconference software where participants can use their own personal devices, reduced potential barriers to implementation related to purchasing equipment and software. Participants also found the convenience and flexibility in the booking time to be an enabler. The structure of telehealth sessions allowed them to fit treatments around their busy schedule. The length of the telehealth sessions was important for our trial to assess exercise adherence and fidelity during these sessions. Shorter videoconference checks of competency (e.g. a check of 1 set only) may improve acceptability for some individuals. Regular contact and assurance provided by physiotherapist improves the therapeutic relationship.

However, some participants expected ‘hands on’ assessment (provided at baseline in our trial) which could be a potential barrier to fully remote management for Achilles tendinopathy. This expectation is inconsistent with current evidence that suggests that the use of telehealth is of similar accuracy of assessment compared to face-to-face when diagnosing the majority of ankle disorders [23] and musculoskeletal conditions [24, 25]. Participants are found to have similar concerns around the validity of diagnosing red flags or more serious pathology during a musculoskeletal assessment [22]. Combining face-to-face with telehealth follow-ups (as in our trial) may alleviate some of these concerns. Consistent with this, few physiotherapists felt it was slower to develop a therapeutic alliance via videoconference compared to standard care. Lack of physical contact that facilitates therapeutic alliance may have impacted this. Providing adequate condition-specific guidelines could address such concerns in future research.

Poor internet network was identified as extrinsic barrier for both participants and physiotherapists in our study. Poor internet connection could preclude physiotherapists’ assessment during the daily practice. Adequate internet bandwidth and data security are critical factors to effectively deliver internet-based telehealth approaches [26]. Studies that examine patient satisfaction with telehealth, defined technical difficulties as the most common area of dissatisfaction [27, 28]. This barrier did not impact on participants’ acceptability of the services received as the communication was either re-established or the appointment rescheduled.

### Gym-based exercise barriers and enablers

The overall adherence to gym-based exercise that we observed in our trial was poor, ranging from 49 to 68%. This may be explained by some of the barriers reported in this qualitative study. The time commitment to undertake the exercise in a gymnasium (three times per week) was a challenge especially if the number of the Smith machines was limited. This may adversely affect participation as predicted by the physical activity maintenance theory [29]. It includes two environmental variables: life stress and physical activity environment. These environmental barriers we identified were largely concordant with previous studies examining factors affecting health care participation in this Achilles tendinopathy population [30, 31] and may also explain the variable exercise adherence that we observed in our trial. Home exercise may have reduced the time and travel requirements and increased adherence. There is evidence to suggest that some adults with musculoskeletal conditions prefer to exercise at home for reasons of convenience, privacy, incorporation into daily routines, and diminished access to transport [22, 32, 33].

Although participants in our study did not report any maladaptive beliefs such as fear-avoidance, they expressed concern towards the nature of the exercises where they reported discomfort from the pressure of Smith bar while exercising with weight. This observation needs to be carefully addressed in future trials as it could be a potential deterrent to adherence or progression (performing the required load-intensity). Similar loading principles could be applied using different equipment such as leg press or free weight which could reduce discomfort on the thigh and could also eliminate the frustration of having to wait for particular exercise equipment.

There were multiple enablers to the exercise reported but they did not appear to offset the barriers given the unsatisfactory adherence to exercise in our trial. First, improvement in participants pain and physical function provided motivation to exercise. Substantial early benefit was seen at the six-week time point which may have motivated participants to persevere with the interventions. This is consistent with the health beliefs model where the perceived benefits of an intervention predicts the likelihood of engaging in the associated intervention behaviours [34]. Second, the regular contact with a physiotherapist via videoconference enabled feedback and reassurance such as appropriate load with each exercise and the correct technique. Regular contact with a physiotherapist compared to use of an exercise brochure has previously been found to be associated with increased adherence and fidelity to home-based exercise program in middle-aged adults with musculoskeletal pain [35]. A greater adherence was found to a home-based falls prevention program among older adults (≥ 60 years) when the program provided at least twice weekly by a physiotherapist compared to non-health provider [36]. Third, prescribing only two exercises was viewed as an enabler. Total number of exercises per session has been shown to predict adherence to home-based exercise among participants with musculoskeletal pain (i.e. three or fewer was predictive) [37]. The concept of providing simple exercises to improve self-efficacy and assist with adherence levels is outlined in the Health Beliefs Model [34] and recommended in expert consensus for various musculoskeletal conditions [38, 39]. Fourth, providing individualised advice about acceptable pain seemed to be important information that facilitated self-manage. Emphasising that pain during rehabilitation can be normal and acceptable can improve adherence in the rehabilitation and influence the extent to which the individuals will persevere when faced with challenges or distractions [32].

### Strengths and limitations

The strength of this study lies in gaining in depth assessment of both the participants and physiotherapists perspectives in gym-based exercise intervention for Achilles tendinopathy that included a telehealth component. The data were collected and analysed by researchers who were independent to the trial in order to reduce intellectual bias. A limitation of this study was the sample size of participants. Although we reached data sufficiency, themes may not represent the perceptions or conclusive evidence of all participants in the primary trial. Another limitation may be selection bias because participants who agreed may have had some positive feelings towards telehealth or gym rehabilitation to start with, despite many not having gym memberships.

## Conclusions

Gym-based exercise intervention involving weekly telehealth monitoring for individuals with Achilles tendinopathy was acceptable for both participants and physiotherapists. Potential enablers and barriers to adherence with telehealth sessions and exercise interventions were identified and findings can be used to guide further research and clinical practice in this emerging area.

## Supplementary Information

**Additional file 1:.** Interview questions guide for participants

**Additional file 2:.** Interview questions guide for physiotherapists

## Data Availability

The datasets used and/or analysed during the current study are available from the corresponding author on reasonable request.

## Abbreviation

VISA-A: Victorian Institute of Sports Assessment – Achilles questionnaire
