# Supplementary material for: Telehealth sounds a bit challenging, but it has potential: participant and physiotherapist experiences of gym-based exercise intervention for Achilles tendinopathy monitored via telehealth
Source: BMC Musculoskelet Disord. 2021 Feb 4;22:138. doi: 10.1186/s12891-020-03907-w (PMC7860049; doi:10.1186/s12891-020-03907-w)
Supplement: Supplementary file 1 — Additional file 1:. Interview questions guide for participants [file 12891_2020_3907_MOESM1_ESM.docx]

**Additional file 1.** Interview questions guide for patients

| **PARTICIPANT INTERVIEW QUESTIONS** | **PROMPT** |
| --- | --- |
| What made you join the study? | Was anyone else involved in the decision? How did this impact your decision?  What were your hopes of being involved in this study?  How did you feel about using Zoom? |
| What was your experience of the exercises prescribed to you? | What was it like having treatment via zoom?  What was important to you?  What was good about the gym experience? What was bad “or not ideal”?  Did you feel the exercises were relevant? Expand  How was the telehealth session you did with your PT?  How were the sessions you did on your own?  Was there a difference? Expand  What were your thoughts on what was prescribed e.g. the intensity, repetition, and number of sets in relation to the length of the treatment |
| How did you find the education material we have provided to for you?  1:1 – exercise prescription and training | Was it enough?  Any comments? |
| Has the intervention changed the way you view your Achilles? | Did your Achilles pain/ activity level change? How? |
| Did you do all the 3 sessions each week? | Why? Why not?  Expand on barriers e.g. work commitments, family? gym environment itself? |
| What was your experience working with PT over the zoom? | Was it tricky?  Could we do anything to make it better?  What would you suggest?  Would you recommend others to participate in using Zoom physiotherapy consultations for Achilles pain?  What are your thoughts towards using Zoom for physiotherapy consultations in the future? |
