# Supplementary material for: Telehealth sounds a bit challenging, but it has potential: participant and physiotherapist experiences of gym-based exercise intervention for Achilles tendinopathy monitored via telehealth
Source: BMC Musculoskelet Disord. 2021 Feb 4;22:138. doi: 10.1186/s12891-020-03907-w (PMC7860049; doi:10.1186/s12891-020-03907-w)
Supplement: Supplementary file 2 — Additional file 2:. Interview questions guide for physiotherapists [file 12891_2020_3907_MOESM2_ESM.docx]

**Additional file 2.** Interview questions guide for physiotherapists

| **PHYSIOTHERAPISTS INTERVIEW QUESTIONS** | **PROMPT** |
| --- | --- |
| What made you join the study? | What is your experience of dealing with people with Achilles pain?  Was anyone else involved in your decision?  How did you feel about using a zoom intervention? |
| What is your opinion on the role of exercise fidelity in Achilles tendinopathy? |  |
| Tell me about your experiences using Zoom for your physiotherapy weekly supervised session. | What was it like providing treatment/information advice/instructions over Zoom?  What was important to you?  What was good about it?  What was bad about it? What could be improved? |
| How did you find the education material we have provided to you?  1:1, online– exercise prescription and training | Was it enough?  Was the material different in any way to the education content you would normally provide patients?  If yes, in what way?  How do you know if your patient has understood your education? |
| How well do you think your patients understood the instructions you prescribed over zoom? | Were you confident that your patients could perform the exercises safely and effectively on their own? Expand |
| I would like you to reflect on your communication with your patients and the relationships you developed. What are your thoughts about this? | How was the session with your patients?  Did the zoom impact your relationship? |
| What do you think the main outcomes were for the patients from this physiotherapy program? | Pain and function? |
| Do you think a supervised physiotherapy session is something you will use with your patients? | Do you see any potential issues? |
